# *Thesium ebracteatum* (Santalaceae) rediscovered in Romania: ecological and biogeographical context

**DOI:** 10.3897/BDJ.13.e172455

**Published:** 2025-10-29

**Authors:** Jan Roleček, Jan Novák, Přemysl Bobek

**Affiliations:** 1 Department of Botany and Zoology, Faculty of Science, Masaryk University, Brno, Czech Republic Department of Botany and Zoology, Faculty of Science, Masaryk University Brno Czech Republic; 2 Department of Paleoecology, Institute of Botany of the Czech Academy of Sciences, Brno, Czech Republic Department of Paleoecology, Institute of Botany of the Czech Academy of Sciences Brno Czech Republic; 3 Department of Botany, Faculty of Science, Charles University, Prague, Czech Republic Department of Botany, Faculty of Science, Charles University Prague Czech Republic; 4 Department of Paleoecology, Institute of Botany of the Czech Academy of Sciences, Průhonice, Czech Republic Department of Paleoecology, Institute of Botany of the Czech Academy of Sciences Průhonice Czech Republic

**Keywords:** endangered species, floristic record, forest-steppe grassland, habitat conservation, past land use, relict species, Romania, vegetation composition

## Abstract

**Background:**

*Thesium ebracteatum* Hayne is an endangered hemiparasitic plant, rare in the south-western part of its range. It is listed in Annex II of the EU Habitats Directive and appears on the national Red Lists of many European countries. In Romania, the species is considered extinct according to the national Red List, with only a few historical records from Transylvania.

**New information:**

In June 2025, approximately 100 individuals of *T. ebracteatum* were discovered near Bălcăuți, Suceava Region, Romania. This represents the first national record of the species in several decades and the first ever from eastern Romania. The species occurred in a stand documented by a vegetation plot, corresponding to the phytosociological association *Brachypodio pinnati*-*Molinietum arundinaceae*. The isolated occurrence near Bălcăuți, similar to other peripheral populations in the south-western part of the species’ range, is regarded as relict. This interpretation is further supported by the disjunct occurrence of other rare plant species typical of peri-Carpathian forest-steppe. Historical maps indicate that the site once formed part of an extensive grassland complex known as Horaiț/Horaiza, most of which was converted to arable land during the 19^th^ and 20^th^ centuries. For the long-term conservation of *T. ebracteatum* and other rare species at the site, targeted management is required, including the prevention of further ploughing and the extension of legal protection to all valuable local stands.

## Introduction

*Thesium ebracteatum* (Santalaceae), like other European representatives of the genus, is a short-statured hemiparasitic plant with narrow leaves and small white flowers (Tutin 1993). The inconspicuous appearance, however, cannot hide other notable characteristics of the species, such as specific habitat requirements and characteristic distribution pattern (Hendrych 1969).

The range of *T. ebracteatum* is continental, centred in the subboreal (Sarmatian) part of the temperate belt, extending from northern Germany and formerly Denmark to the Ural Mts in European Russia (Hendrych 1969, GBIF Secretariat 2025). While the species is relatively abundant in the north-eastern part of its range, its occurrence in the southwest is fragmented and frequently associated with refugial sites, such as fen meadow complexes (Čeřovský 1999). Throughout its range, *T. ebracteatum* prefers slightly moist to slightly dry oligotrophic grasslands, forest margins and open-canopy forests (Dostálek and Münzbergová 2010, Shevchyk et al. 2023).

The rarity of the species in the south-western part of its range is reflected in its conservation status. Although globally classified as Least Concern (IUCN 2025), *T. ebracteatum* is listed in Annex II of the EU Habitats Directive (Council Directive 92/43/EEC) and is included in the national Red Lists of many European countries. It is assessed as Critically Endangered in Austria (Schratt-Ehrendorfer et al. 2022), the Czech Republic (Grulich 2017), Germany (Metzing et al. 2018) and Latvia (Andrušaitis and Vimba 2003), Endangered in Lithuania (Rašomavičius 2021) and Vulnerable in Estonia (Kull et al. 2018) and Poland (Kaźmierczakowa et al. 2016). Moreover, it is classified as Probably Extinct in Italy (Rossi et al. 2013) and Extinct in Denmark (Moeslund et al. 2023) and Slovakia (Eliáš et al. 2015); however, in recent years, several populations have been discovered in Slovakia (Jasík and Dítě 2017, iNaturalist 2025).

In Romania, the historical occurrence of *T. ebracteatum* has been reported from several sites in Transylvania, including Cluj-Napoca, Becaș, Ragla and Mediaș (Oprea 2005, Sârbu et al. 2013). The former (Oprea 2005) classified the species as rare and data deficient, whereas the latter (Sârbu et al. 2013) considered its occurrence uncertain. In the national Red List (Oltean et al. 1994), the species is listed as extinct. Old herbarium voucher specimens from Becaș (Cluj County), collected by Wolff, and from Ragla (Bistrița-Năsăud County), collected by Porcius, are housed in the herbarium of Babeș-Bolyai University in Cluj-Napoca (M. Pușcaș & A. Indreica, pers. comm.).

Here, we report a recent record of *T. ebracteatum* from eastern Romania, describe vegetation and habitat conditions of its site and discuss the biogeographical context and suitable conservation management for the species.

## Materials and methods

### Study area

**Figure 1.**
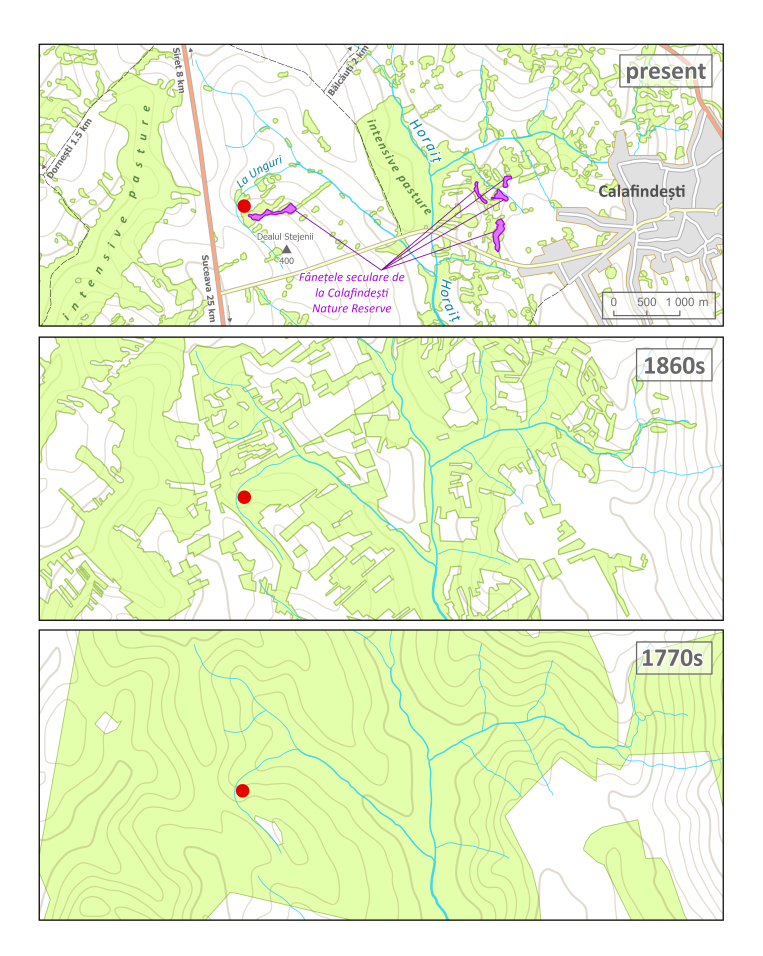
Map of the study area. The red dot denotes the locality of *T. ebracteatum*. Green shading represents the distribution of grasslands at present (top) and during two historical periods: the 1860s (middle), based on the maps of the Austrian Second Military Survey, and the 1770s (bottom), based on the maps of the First Military Survey. Present-day grasslands include both permanent grasslands and grass and herb-rich fallows.

The study area is in north-eastern Romania, between the villages of Bălcăuți, Dornești and Calafindești, approximately 25 km northwest of the City of Suceava (Fig. 1). It consists of a small remnant of steppe and wet grassland, surrounded by an intensively used agricultural landscape. The local topography is shaped by a protruding low ridge, Dealul Stejenii (400 m a.s.l.; also called La Stejari), which slopes southwestwards and northwards towards the shallow valley of the La Unguri stream (370 m a.s.l.; now more of a drainage channel), within the catchment area of the Horaiț River, a tributary of the Suceava River (Țibu 1997). To the east, the ridge abruptly transitions into undulating slopes with landslides. The bedrock consists predominantly of fine-grained calcareous marine sediments of Miocene age (Volhynian stage; Geological Institute of Romania (2025)). Climatic conditions (Karger et al. 2017) correspond to the altitude (mean annual temperature 8.4℃) and proximity of the Eastern Carpathian Mountains, which promote somewhat higher rainfall (mean annual precipitation close to 660 mm).

The site represents a remnant of the once extensive meadows (Fig. 1, Arcanum (2025a), Arcanum (2025b)), whose botanical value was already recognised in the mid-19^th^ century (Herbich 1859). In the late 19^th^ century, the prominent Romanian botanist Aurel Procopianu-Procopovici conducted a detailed analysis of a large remnant of these meadows between the villages of Bălcăuți and Românești, to which he referred as the Horaiza ancient meadows (Procopianu-Procopovici 1893). Today, several scattered fragments are protected under the Fânațele seculare de la Calafindești Nature Reserve, which extends close to our study area. Although some sources report the size of the Reserve to be as great as 17.3 hectares, its actual boundaries vary amongst sources (Mânzu et al. 2019). According to current maps of protected areas, the stand hosting the population of *T. ebracteatum* reported here is not under legal protection (Fig. 1, European Environment Agency (2025), Ministerul Mediului, Apelor și Pădurilor (2025)). However, it forms part of a more or less continuous area with well-developed grassland vegetation, approximately 5 hectares in extent, located within the cadastral territories of the municipalities of Calafindești and Bălcăuți. Roughly half of it lies within the Nature Reserve.

### Vegetation survey

Vegetation data were recorded in a 4 × 4 m plot using standard phytosociological methodology, with the extended Braun-Blanquet scale used for species cover-abundance estimation (Dengler et al. 2008). Taxonomic concepts and nomenclature of vascular plant taxa follow Euro+Med PlantBase (Euro+Med 2025), except for *Ligularia glauca* (L.) O. Hoffm. (incl. *L. carpathica*) and other broadly conceived taxa: *Knautia arvensis* agg. (*K. arvensis*, *K. kitaibelii*), *Pulmonaria mollis* agg. (*P. dacica*, *P. mollis*), *Veronica chamaedrys* agg. (*V. chamaedrys*, *V. vindobonensis*) and *Vicia cracca* agg. (*V. cracca*, *V. tenuifolia*).

### Past land use

To illustrate the historical context and the dynamics of the retreat of local forest-steppe grasslands as a potential habitat of *T. ebracteatum*, we mapped grassland extent as depicted on maps of the Austrian First Military Survey (1773–1775; Arcanum (2025a)), the Second Military Survey (1861–1864; Arcanum (2025b)) and the contemporary Copernicus High-Resolution Layer Grasslands, reference year 2021 (European Environment Agency 2024).

## Data resources

### Recorded vascular plant species

A machine-readable list of vascular plant species recorded together with *T. ebracteatum* is provided (Suppl. material 1).

## Taxon treatments

### Thesium ebracteatum

Hayne

3ACB2327-6A0E-5CF9-9367-EC95A51ACA4F

#### Distribution

**Figure 2.**
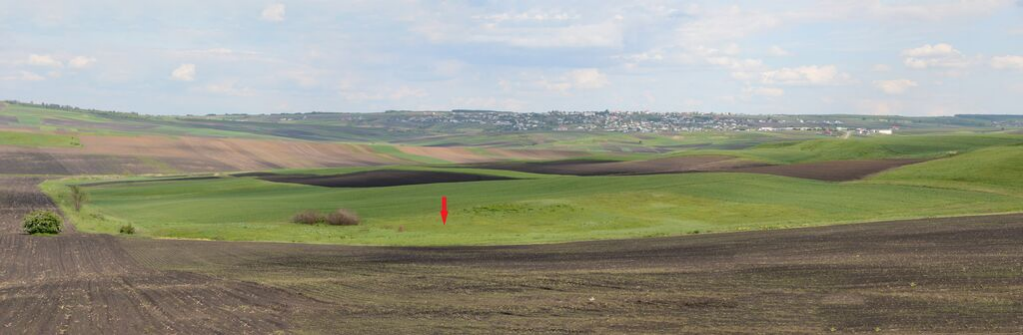
An overall view of the *Thesium ebracteatum* site. The red arrow indicates the location of the population. Fânațele seculare de la Calafindești Nature Reserve is shown as green slopes on the right side of the image, just east of the locality. The horizon is dominated by the village of Calafindești.

**Figure 3.**
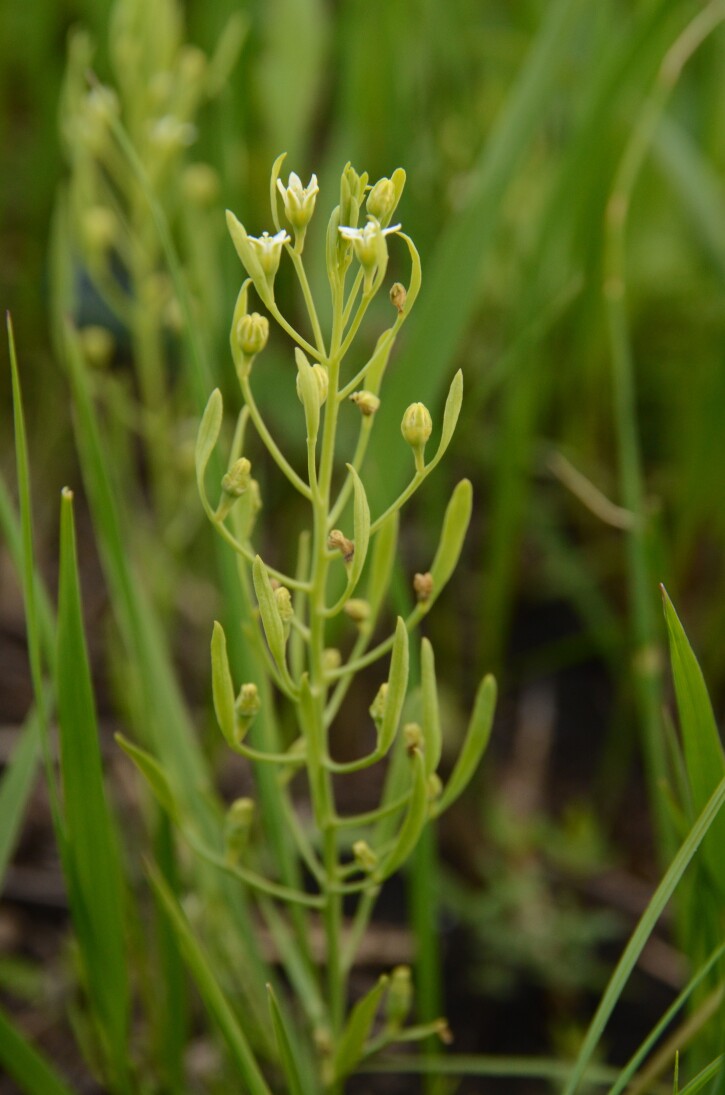
*Thesium ebracteatum* at the newly-found locality. The species is characterised by a tuft of sterile leaves at the top of the stem, the absence of bracteoles (only a single bract subtending each flower is present), shortly pedicellate flowers and short perianth segments that, in fruit, are, at most, as long as the nutlet.

*Thesium ebracteatum* was recorded on 14 June 2025 near Bălcăuți (Suceava Region), at the foot of Dealul Stejenii Hill, in a transitional zone between species-rich forest-steppe grassland and intermittently wet *Molinion* meadow (Fig. 2). Approximately 100 individuals were found here. The observed plants were characterised by a tuft of sterile leaves at the top of the stem, the absence of bracteoles, shortly pedicellate flowers and short perianth segments that, in fruit, were, at most, as long as the nutlet (Fig. 3). *Thesium rostratum*, a similar Central European species reported as insufficiently known from Romania (Oltean et al. 1994), is differentiated by its sessile flowers, a tubular rather than campanulate perianth that is up to three times as long as the nutlet in fruit and a rhizome lacking stolons (Tutin 1993). A herbarium voucher specimen from the newly-discovered population of *T. ebracteatum* has been deposited in the Herbarium of Masaryk University, Brno (BRNU).

#### Notes

##### Vegetation

A phytosociological relevé of local vegetation with *T. ebracteatum* was recorded the following day.

Relevé 1: Romania, Suceava Region, Bălcăuți, foot of the Dealul Stejenii Hill, forest-steppe grassland transitioning to intermittently wet *Molinion* meadow, latitude 47°51'25.6"N, longitude 26°03'19.8"E (WGS-84), altitude 375 m a.s.l., plot size 16 m^2^, slope inclination 5°, slope aspect 330°, cover of herb layer 65%. Date: 15 May 2025. Author: J. Roleček.

*Inula salicina* 3, *Carex montana* 2b, *Potentilla alba* 2a, *Serratula tinctoria* 2a, *Filipendula vulgaris* 1, *Molinia arundinacea* 1, *Peucedanum cervaria* 1, *Anemone nemorosa* +, *Brachypodium pinnatum* +, *Campanula glomerata* +, *Campanula persicifolia* +, *Cirsium pannonicum* +, *Clematis recta* +, *Colchicum autumnale* +, *Crepis praemorsa* +, *Euphorbia angulata* +, *Festuca rupicola* +, *Festuca valesiaca* +, *Galium boreale* +, *Galium verum* +, *Geranium sanguineum* +, *Inula hirta* +, *Iris graminea* +, *Knautia arvensis* agg. +, Lathyrus pannonicus subsp. collinus +, *Lathyrus pratensis* +, *Plantago media* +, *Potentilla erecta* +, *Primula veris* +, *Prunella grandiflora* +, *Pulmonaria mollis* agg. +, *Ranunculus auricomus* coll. +, *Ranunculus polyanthemos* +, *Salvia pratensis* +, *Silene nutans* +, *Stachys officinalis* +, *Thesium ebracteatum* +, *Trollius europaeus* +, *Veratrum lobelianum* +, *Veratrum nigrum* +, *Vicia cracca* agg. +, *Viola hirta* +, *Centaurea jacea* r, *Dianthus superbus* r, *Galium album* r, *Muscari botryoides* r, *Phragmites australis* r, *Poa angustifolia* r, *Sanguisorba officinalis* r, *Succisa pratensis* r, *Tanacetum corymbosum* r, Taraxacum sect. Taraxacum r, *Thalictrum aquilegiifolium* r, *Trifolium montanum* r, Valeriana stolonifera subsp. angustifolia r, *Veronica chamaedrys* agg. r, Viola canina subsp. ruppii r.

## Discussion

### Significance of the find

According to the available information, our find of *T. ebracteatum* is the first record of a species considered extinct in Romania (Oltean et al. 1994) after many decades. At the same time, it is the first record from the eastern, pre-Carpathian part of Romania, from where the species had not been previously reported in literature. However, it has been reported from the northern part of neighbouring Moldova (Geydeman 1975). Based on the habitat conditions and biogeographical context summarised below, we believe that the occurrence of *T. ebracteatum* near Bălcăuți is natural and that this rare and inconspicuous species has so far been overlooked. This view is further supported by our own experience, as, during one of our previous visits, we misidentified the species as *T. linophyllon*. We suggest that, according to the IUCN rules (IUCN 2012), *T. ebracteatum* now qualifies as Critically Endangered under criteria B1ab(iii)+2ab(iii) in Romania. The assessment is based on its extremely limited extent of occurrence and area of occupancy, the occurrence of only a single population and a continuing decline in habitat quality.

### Habitat conditions

At the newly-discovered site, *T. ebracteatum* grows at the transition between moderately dry and moderately wet species-rich grassland. It does not extend into the drier grasslands (*Polygalo*-*Brachypodietum* association), where it is replaced by *T. linophyllon*, nor into the typical vegetation of intermittently wet meadows (*Molinion caeruleae* alliance) or more moisture-demanding vegetation. This narrow ecological niche may be related to suboptimal climatic conditions at the periphery of the species’ range; on the other hand, the species occurs in quite similar vegetation in other parts of its distribution area as well (Hendrych 1969, Dostálek and Münzbergová 2010, Shevchyk et al. 2023).

The species composition of the stand with *T. ebracteatum* corresponds well to the phytosociological association *Brachypodio pinnati*-*Molinietum arundinaceae*. This widely scattered type of forest-steppe grassland is characteristic of the Carpathian periphery and reaches its eastern distributional limit in the Suceava Region (Roleček 2023, Roleček et al. 2025).

### Biogeographical relationships

Although the newly-discovered site of *T. ebracteatum* is remote and peripheral within the species’ range, it is consistent with the overall biogeographical pattern of the species. As previously mentioned, *T. ebracteatum* has a number of isolated sites on the south-western edge of its range, typically in refugial habitats where it likely survives as a relict from a time of wider distribution. We suggest that the occurrence near Bălcăuți is also of relict origin. This interpretation is supported by the presence of several other biogeographically significant species with marginal and isolated occurrences in the study area.

Particularly, the presence of the last remaining lowland population of *Ligularia glauca* in Romania is well known (Oprea 2005, Mânzu et al. 2019). *Ligularia glauca* is an important continental element and likely a glacial relict with its main distribution in the mountains of southern Siberia (Koczwara 1927, Horsák et al. 2015). Amongst the species with a large continental disjunction belongs also *Iris ruthenica*, which occurs at the study site as well and has several populations in well-preserved forest-steppe grasslands in the broader region (Oprea 2005, personal observation). Several other rare species occur here, such as *Pulsatilla patens* and *Adenophora liliifolia*, which, like *T. ebracteatum*, are subboreal (Sarmatian) forest-steppe elements (Tomescu 2016, personal observation). An additional characteristic is the presence of light-demanding yet mesophilous to hygrophilous species with boreal or montane affinity, such as *Salix starkeana*, *Thalictrum aquilegiifolium*, *Trollius europaeus* and *Veratrum lobelianum* (Tomescu 2016, personal observation). Their presence is probably supported by the relatively humid climate of the region, which may have contributed to their long-term persistence, so that migration from the nearby Carpathians may not have played a key role in their presence. As a result, a specific vegetation developed in which forest-steppe species (e.g. *Dictamnus albus*, *Festuca valesiaca*, *Polygala major*, *Tephroseris integrifolia*) intermingle with more hygrophilous ones.

### Peri-Carpathian forest-steppe

Such species combinations are typical of the peri-Carpathian forest-steppe, a relict ecosystem occurring along the periphery of the Carpathians in places with forest climate and a long-term continuity of open, treeless habitats (Roleček 2023). Compared to drier types of forest-steppe, mesophilous to hygrophilous species and vegetation types are more abundant here, while narrow-leaved dry grasslands and salt marshes are absent or rare. The affiliation of vegetation of the study site to the peri-Carpathian forest-steppe is further supported by the presence of tall herb-rich stands of the association *Trollio*-*Clematidetum recti* (*Geranion sanguinei* alliance; Roleček et al. (2022)). It is confined to the most productive places within the steppe grasslands (depressions, footslopes) and the stands protected in the Fânațele seculare de la Calafindești Nature Reserve rank amongst the most luxuriant and floristically rich within the distribution range of this peculiar vegetation type.

According to the results of our palaeoecological research (Roleček 2023, Novák et al. 2024), the peri-Carpathian forest-steppe is derived from the hemiboreal forest-steppe of the Late Glacial and Early Holocene, which is consistent with the biogeographical affinities of *T. ebracteatum*. Already the monographer of the genus *Thesium* R. Hendrych assumed that the present distribution of the species is a remnant of early postglacial formations of light taiga forests, humid and swampy primary meadows and cooler steppe (Hendrych 1969). The archaic character of meadow vegetation in the study area was recognised earlier by A. Procopianu-Procopovici, although he did not specify its age or developmental relationships (Procopianu-Procopovici 1893). Long-term persistence of local grassland ecosystems is supported also by the abundance of deep chernozem-like soils (especially phaeozems), while more recently deforested areas are dominated by luvisols (FAO and IIASA 2022).

### Historical, present and suggested management

Historical maps indicate that the study area was formerly part of an extensive grassland complex. By the late 19^th^ century, the remaining fragments of these grasslands were used as hay meadows and were also regularly burnt to prevent shrub encroachment. Grazing (e.g. by sheep) occurred primarily near villages; however, the species composition of grazed grasslands was different (Procopianu-Procopovici 1892, Procopianu-Procopovici 1893).

Most of the local grasslands were ploughed during the 19^th^ and 20^th^ centuries and the fertile phaeozem soils were used for cultivating maize, clover and other crops (Procopianu-Procopovici 1892, Procopianu-Procopovici 1893, Țibu 1997). Currently, mowing is only carried out on a small scale, but burning is still practised fairly regularly outside the growing season (during our visits in 2017 and 2025, the soil surface showed clear signs of burning). Extensive grassland tracts between Calafindești and Dornești are subject to intensive grazing, which has a largely negative impact on the conservation of natural values.

The current low-input management may suffice only in the short term; for the long-term conservation of populations of valuable species, targeted conservation management is needed. This should include annual mowing, possibly combined or alternated with extensive grazing and prescribed off-season burning. A key task for governmental and local authorities is to prevent further ploughing of grasslands and to extend legal protection to all valuable stands, including the one hosting *T. ebracteatum*. It would also be beneficial to establish a grassy buffer to protect the site from the adverse effects of the surrounding agricultural fields and perhaps also to restore the La Unguri stream to prevent excessive drainage of the area. In addition to protection, it is essential to thoroughly investigate the biodiversity in the best-preserved grasslands in order to provide information for and prioritise conservation efforts.

## Supplementary Material

XML Treatment for Thesium ebracteatum

6614FEA0-9DEC-59F9-90CE-C2E9576C4437 10.3897/BDJ.13.e172455.suppl1 Supplementary material 1 List of vascular plant species recorded together with Thesium ebracteatum Data type Occurrences Brief description Species list corresponds to the phytosociological relevé provided in the paper. File: oo_1413953.csv https://binary.pensoft.net/file/1413953 Jan Roleček
